# Editorial Note: Host-Associated Metagenomics: A Guide to Generating Infectious RNA Viromes

**DOI:** 10.1371/journal.pone.0350242

**Published:** 2026-05-27

**Authors:** The PLOS One Editors

The concerns underlying the Expression of Concern on this article [1,2] have been dismissed.

PLOS investigated this article [1] as one of a series of submissions for which there were research ethics concerns. During editorial follow-up, PLOS determined that [1] meets PLOS’ ethics requirements and no corrective action is warranted for [1] at this time.
